# Inflammatory reaction to fish oil coated polypropylene mesh used for laparoscopic incisional hernia repair: a case report

**DOI:** 10.1186/s12893-016-0123-8

**Published:** 2016-02-11

**Authors:** Chia Yew Kong, Lee Lee Lai, Amanda Yin Yen Khoo, Nazarina Abdul Rahman, Kin Fah Chin

**Affiliations:** School of Medicine, University of Glasgow, Wolfson Medical School Building, University Avenue, Glasgow, G12 8QQ Scotland, UK; Department of Nursing Science, University of Malaya, Lembah Pantai, 59100 Kuala Lumpur, Malaysia; The University of Queensland Mayne Medical School, 288 Herston Road, Herston, Brisbane, QLD 4006 Australia; Redland Hospital, Weippin Street, Cleveland, QLD Australia; Department of Pathology, University of Malaya, University Malaya Medical Centre, Lembah Pantai, 59100 Kuala Lumpur, Malaysia; Department of Surgery, University of Malaya, University Malaya Medical Centre, Lembah Pantai, 59100 Kuala Lumpur, Malaysia; Department of Surgery, Tunku Abdul Rahman University, Sungai Long Campus, Jalan Sungai Long, Bandar Sungai Long, Cheras, 43000 Kajang, Selangor Malaysia

**Keywords:** Hernia, Fish oil coated hernia mesh, Hernia repair complications, Hernia mesh

## Abstract

**Background:**

Polypropylene meshes are widely used in hernia repairs. Hernia meshes have been developed incorporating coatings of active agents. One commercially available mesh has a fish oil coating which is promoted as having anti-inflammatory properties. We report a case, a symptomatic foreign body granuloma reaction associated with a fish oil coated polypropylene mesh, which required eventual mesh explantation.

**Case presentation:**

A 61-year old lady with previous peptic ulcer disease underwent a laparoscopic intraperitoneal placement of mesh for incisional hernia utilising a fish oil coated polypropylene mesh. The patient presented 3 months after the procedure complaining of dyspepsia and pain at the operative site. There was no discharge. The patient was managed conservatively. She presented 10 months post-operatively with progressively worsening symptoms and a hard palpable mass in the epigastrium. Abdominal laparoscopy revealed dense adhesive disease around the mesh with exudates. Adhesiolysis, mesh explantation and a partial gastrectomy was performed. Histopathological examination revealed a foreign body granuloma formation to the mesh.

**Conclusion:**

In-vivo studies looking at intraperitoneal mesh placement with fish oil coatings including data on surgical outcomes such as fistula and adhesive characteristics are scarce in the literature. Further monitoring and studies are required to investigate the safety and efficacy profile of this mesh type in in-vivo models.

## Background

Incisional hernias are a common complication after a abdominal surgery, with reported incidence rates ranging from 3 to 20 % [1]. The use of laparoscopy in surgery and laparoscopic intraperitoneal placement of onlay mesh (IPOM) specifically continues to be a matter of debate [1]. Current data indicates that laparoscopic repair of incisional hernias are asssociated with favourable outcomes such as length of stay and infective complications but may be associated with worse cosmetic outcomes [1].

The development of laparoscopic approach presents challenges as direct contact of a mesh with intraperitoneal organs may provoke an inflammatory response which in turn may lead to to adhesion and fistula formation [2]. The C-Qur™ Mesh (Atrium Medical, Hudson, NH, USA), is a polypropylene mesh coated with highly purified pharmaceutical grade fish oil comprising a blend of triglycerides and Omega 3 fatty acids [3]. This mesh belongs to a family of meshes developed with coatings that aim to possess favourable anti-adhesion properties when in contact with intraperitoneal organs.

## Case presentation

A 61-year-old Chinese lady presented with a 6-month history of a localised abdominal swelling and distention one and a half years post-midline laparotomy for a bleeding peptic ulcer. Her co-morbidities include a mild hiatus hernia and ischaemic heart disease for which she was on a low-molecular-weight heparin and a platelet inhibitor. She is American Society of Anaesthesiologists (ASA) Grade II and is not on regular non-steroidal, anti-inflammatory medications. On examination, an incisional hernia was found at the previous laparotomy site. It had a smooth surface and a positive cough impulse.

The patient underwent a laparoscopic intraperitoneal placement onlay mesh repair for her hernia. An Optic view port (12 mm) was placed at the left subcostal region as primary port entry with two 5 mm ports at the left flank. Dense abdominal adhesions were found with small bowel adherent to the anterior abdominal wall within the hernia sac. These were localised to the hernia site.

A laparoscopic adhesiolysis was done dissecting the adherent small bowel off the anterior abdominal wall. The hernia defect measured 16 cm by 20 cm. An 20.32 cm by 25.4 cm sized polypropylene mesh coated with omega 3 fatty acids (fish oil) was placed tension-free with no primary suturing and fixed with two rows of ProTack™ 5 mm titanium helical fasteners to restore the integrity of the wall. The hernia sac was not excised. A standard double crown technique with stay sutures was employed. There were no iatrogenic injuries during adhesiolysis. The patient recovered and the immediate post-operative period was uneventful.

During a routine follow-up visit 3 months after the procedure, the patient complained of mild dyspepsia, intermittent abdominal pain. There was no discharge from the incision site. On examination, there was slight tenderness over the incisional hernia site but no obvious recurrence. The patient was reassured and managed conservatively.

**Fig. 1:**
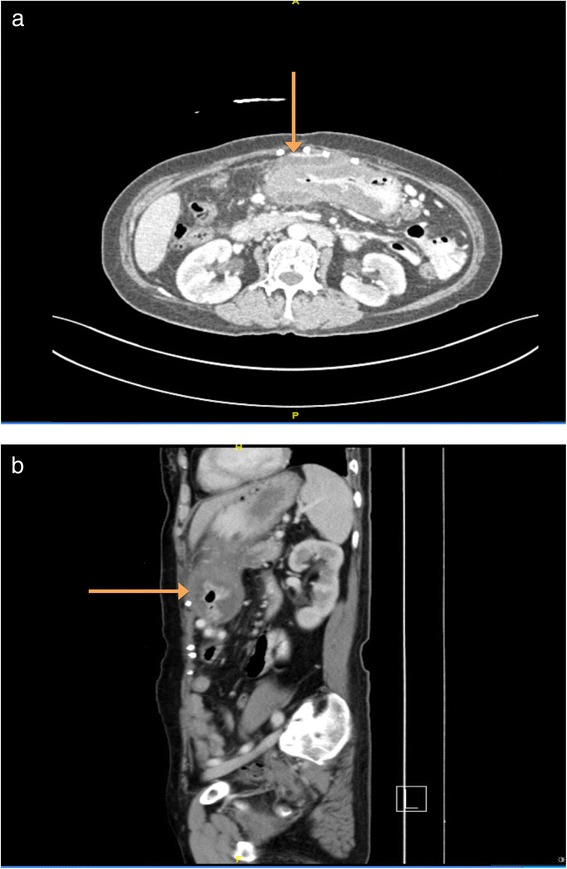
(**a** and **b**) CT imaging (transverse and sagittal views) showing thickening of the body and pylorus of the stomach (orange arrow) and a thin soft tissue plane separating the stomach lesion and the pancreas. The appearance of tissue extending between the two abdominal wall tacks suggested a recurrence of the incisional hernia but this was found not to be the case intraoperatively. Intraoperatively, the mesh was found to be adherent to a loop of small bowel

The patient’s dyspepsia and abdominal pain got progressively worse and a hard tender mass located in the epigastrium was palpated 10 months post-procedure. Computed tomography imaging of the abdomen showed thickening of the body and pylorus of the stomach and a thin soft tissue plane separating the stomach lesion and the pancreas (Fig. 1a, b).

Gastroscopy revealed a thickened gastric antrum. There was no gross evidence of acute recurrence of her ulcer disease. Histopathological examination of biopsies taken from the gastric antrum showed chronic inflammatory granulation and fibrosis with no evidence of malignancy.

The patient subsequently underwent a laparoscopy. Dense intrabdominal adhesions were found intra-operatively. There was an adhesion of the small bowel to the fish oil mesh with fine inflammatory exudates. It was converted to an open procedure. Adhesiolysis, explantation of fish oil coated mesh and ProTack™, partial gastrectomy, gastrojejunostomy and jejuno-jejunostomy were performed. There was no evidence for a recurrence of the incisional hernia. There was no evidence of fistula or chronic sepsis. A sutured repair was undertaken. The condition of the patient improved a few days after the surgery.

**Fig. 2:**
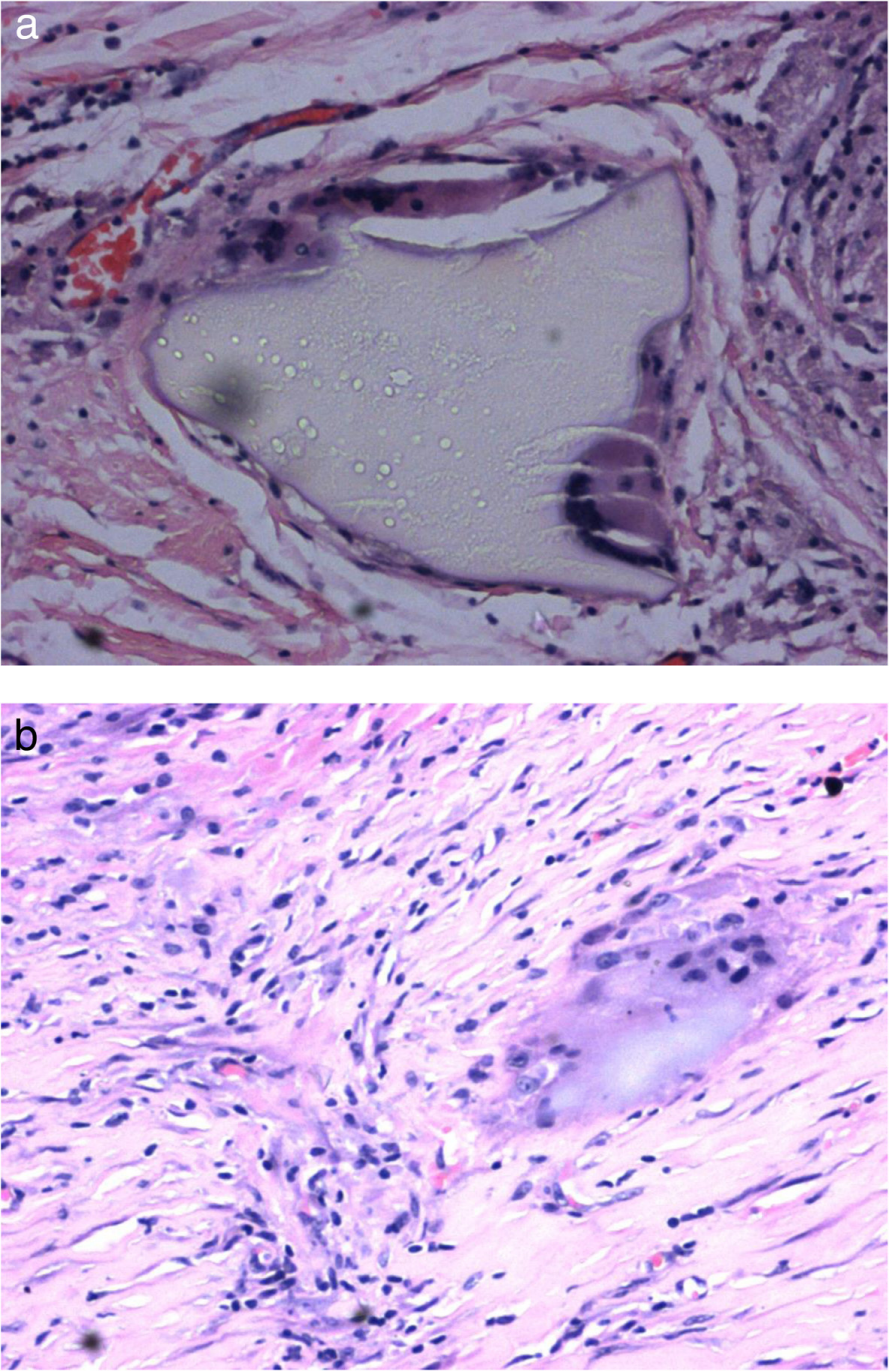
(**a** and **b**) histopathology images showing foreign body granuloma reaction to a pseudomembrane associated with the mesh

Two samples were sent for histopathological analysis: the mesh sample and the partial gastrectomy. Macroscopic analysis of the mesh sample revealed the sample to be a torn piece of plastic mesh embedded in fibrous tissue secured by multiple metal coils. Microscopically, sections of the sample showed partly hyalinised fibrofatty tissue exhibiting an intense foreign-body granulomatous inflammatory reaction towards an amorphous translucent foreign material (Fig. 2a, b).

The partial gastrectomy specimen had thick haemorrhagic fibrous adhesions. The mucosa showed loss of rugae and there appeared to be superficial ulcerations in the mucosa. Microscopically, the gastric specimens revealed submucosal oedema, inflammed granulation and foci of suppurative inflammatory necrosis with extension of the inflammation across the muscularis into the serosa.

The patient had an unremarkable post-operative recovery and has had no further sequelae in subsequent follow-up visits.

## Discussion

The intraperitoneal placement onlay mesh technique involves the placement of mesh within the peritoneal cavity with inevitable contact with adjacent intraperitoneal organs [1]. This contact may provoke an inflammatory reaction between the host tissue and the mesh as a foreign body and may lead to a spectrum of complications from adhesions and fistula formation to bowel obstruction secondary to adhesive disease and organ erosion [2].

These inflammatory reactions are well documented complications of hernia repair with prostheses in both in vitro and in vivo models of hernia repair [2, 4–7]. Specifically, polypropylene mesh is known to cause a non-infective, host-initiated, foreign body granulomatous inflammatory response which may cause scar thickening [4, 8]. This is hypothesised to result from an interaction between native proteins such as fibrinogen and albumin and the mesh’s polymer, causing a denaturation of these proteins which stimulates the formation of a foreign body granuloma, characterised by the aggregration of macrophages and other inflammatory cells [6]. This process can go on for an extended period of time [5, 6].

Hernia meshes have been developed to possess favourable adhesion and anti-inflammatory properties. Mesh materials for intraperitoneal onlay placement have been developed specifically with coatings which have favourable anti-inflammatory properties required in intraperitoneal onlay meshes [9]. The coatings include polyglactin, hyaluronate and cellulose [9]. The mesh used in this patient is a polypropylene mesh coated with pharmaceutical grade fish oil consisting of a blend of triglycerides and Omega 3 fatty acids [3].

Our patient’s initial recovery from the hernia repair was complicated in two ways, [1] an overt, symptomatic foreign body reaction to the mesh material, forming an inflammatory pseudomembrane and [2] a chronic gastritis. There are a number of explanations which could explain her chronic gastritis. The chronic inflammatory changes in the stomach may be due to her underlying previous peptic ulcer disease. It is however unclear whether this chronic inflammatory gastritis could have alternatively been induced by a direct extension of the inflammatory process from the mesh, although the former is a more likely conclusion. The presence of the abdominal wall lesion and gastric disease gave rise to the patient’s complex presentation, with a decision made to perform a laparoscopy to explore these two issues once the patient’s condition deteriorated.

Our case report highlights the development of a foreign body inflammatory reaction to coated mesh. A few in vitro and in vivo studies involving animal models support the anti-inflammatory characteristics of fish oil and C-Qur specifically. Omega-3 fatty acids have been shown to have anti-inflammatory properties [10]. In a rat model study comparing five coated meshes found that C-Qur provoked the least inflammatory reaction amongst the coated meshes [11]. In a rabbit model, no differences were found in adhesion grade and amount between C-Qur and other meshes but C-Qur and a lower percentage coverage of adhesions [12]. There was a single clinical in vivo human study comparing C-Qur with other commercially available meshes in a prospective study of mesh characteristics in patients undergoing laparoscopy who had a previous mesh implant [9, 13]. C-Qur was found to be associated with the lowest adhesion tenacity, least area covered by adhesions and lowest ratio of adhesiolysis time to adhesion area compared to three other meshes [9, 13]. In a rat model comparing the effect of mesh placement across time however, it was found that while C-Qur significantly reduced adhesion formation at 7 days compared to other meshes, this effect was diminished at 30 days with significant increase in adhesions formation [14]. Therefore there is limited clinical data on fish oil coated meshes in humans in the context of intraperitoneal onlay mesh repairs of incisional hernias. Where evidence exists, these are mostly in animal models with a short-term follow-up. Further, these studies have not been replicated to ensure the validity of these findings. Taken together, however, a review of currently available evidence makes this patient’s response to the mesh unexpected. Within the senior author’s experience (Table 1), this is the only patient in the case series of 11 patients with a foreign body reaction who had a mesh explantation.

**Table 1:** The single surgeon’s experience with the C-Qur mesh. Patient 3 is the patient described in this case report. NA indicates missing data

No.	Type of surgery	Duration	Complication	Treatment of complication (if any)	Outcome at 6 months if there was complication
1	Laparoscopic incisional	2 h 30 min	Adhesions and seroma	Adhesiolysis	Recovered, well
2	Laparoscopic left inguinal	1 h 50 min	None		
3	Laparoscopic incisional	NA	Foreign body reaction	Mesh explantation	Recovered, well
4	Laparoscopic incisional	5 h and 35 min	Haematoma		Recovered, well
5	Laparoscopic umbilical	2 h 15 min	Haematoma and wound infection		Recovered, Well
6	Laparoscopic bilateral inguinal	2 h 30 min	None		
7	Laparoscopic incisional	2 h 10 min	None		
8	Laparoscopic bilateral inguinal	2 h and 35 min	None		
9	Laparoscopic paraumbilical	55 min	None		
10	Laparoscopic paraumbilical	NA	Wound and mesh infection	Removal of mesh (infected)	Recovered, well
11	Laparoscopic paraumbilical	NA	Seroma		Recovered, well

There is a caveat when considering the arguments in this case report. It is likely that a number of factors augment the response of the host to the mesh, which includes host and mesh factors, such as her previous peptic ulcer problem.

Our report calls for further research on the safety and efficacy profile of this mesh type. Ongoing prospective monitoring of the use of this mesh type and the incidence of these complications is warranted.

## Conclusion

Polypropylene mesh is known to cause a mild but persistent foreign body reaction but coating with fish oil has been shown to have favourable properties in a few in vivo and in vitro studies, making the findings in this case report unexpected. Data on surgical complications following the usage of fish oil coated mesh for abdominal hernia repair, including infective complications, especially in human studies, however continues to be scarce in the literature. Further studies and monitoring are required to further elucidate the efficacy and safety profile of such meshes.

## Consent

Written informed consent was obtained from the patient’s relative/next-of-kin for publication of this Case report and any accompanying images. A copy of the written consent is available for review by the Editor of BMC Surgery.
